# APOA2-mediated endothelial mesenchymal transition and cancer lipid metabolism reprogramming confers antiangiogenic drug resistance through TGF-β

**DOI:** 10.1038/s41420-026-02984-5

**Published:** 2026-02-27

**Authors:** Su Zhang, Zhou Fu, Fuyi Zhu, Xiaoying Gu, Huaqi Wang, Manqing Cao, Hua Guo, Ti Zhang

**Affiliations:** 1 Department of Gynecologic Oncology, National Clinical Research Center for Cancer, Key Laboratory of Cancer Prevention and Therapy, Tianjin Medical University Cancer Institute and Hospital, Tianjin, China; 2 Department of Tumor Cell Biology, National Clinical Research Center for Cancer, Key Laboratory of Cancer Prevention and Therapy, Tianjin Medical University Cancer Institute and Hospital, Tianjin, China; 3 Department of Hepatobiliary Surgery, National Clinical Research Center for Cancer, Key Laboratory of Cancer Prevention and Therapy, Tianjin Medical University Cancer Institute and Hospital, Tianjin, China; 4 Department of Surgical Oncology, Key Laboratory of Major Diseases in Children, Ministry of Education, Beijing Children’s Hospital, Capital Medical University, National Center for Children’ s Health, Beijing, China; 5 Cangzhou Hospital of Integrated Traditional Chinese and Western Medicine, Hebei, China; 6 Department of Breast Surgery, National Clinical Research Center for Cancer, Key Laboratory of Cancer Prevention and Therapy, Tianjin Medical University Cancer Institute and Hospital, Tianjin, China; 7 Department of Hepatic Surgery, Fudan University Shanghai Cancer Center, Shanghai, China; 8 Department of Oncology, Shanghai Medical College, Fudan University, Shanghai, China

**Keywords:** Liver cancer, Tumour angiogenesis

## Abstract

Angiogenesis is a hallmark of hepatocellular carcinoma (HCC), yet most cases resist antiangiogenic drugs (AADs) targeting VEGFA-VEGFR2, and the molecular mechanisms remain largely unknown. Here, we show that apolipoprotein A2 (APOA2) mediates endothelial-to-mesenchymal transition and reprograms cancer lipid metabolism, inducing AAD resistance in HCC. This occurs via downregulating VEGFR2 in vascular endothelial cells and promoting high cancer cell proliferation with low apoptosis. The whole transcriptome sequencing of unresectable human HCC specimens revealed elevated expression of APOA2 in the AAD-resistant group. Furthermore, the overexpression of APOA2 confirmed resistance to AAD therapy in an HCC-bearing mouse model. AAD treatment had no effect on tumor angiogenesis in HCC overexpressing APOA2, while cancer cells exhibited increased proliferation and reduced apoptosis. Mechanistically, proteomic analysis verified that APOA2 significantly upregulates transforming growth factor-beta (TGF-β) related proteins. Furthermore, the secretion of TGF-β was markedly increased in HCC cell culture medium and the blood of HCC-bearing mice after APOA2 overexpression. On the one hand, TGF-β reduced VEGFR-2 expression and increased mesenchymal gene expression in ECs. On the other hand, TGF-β initiated fatty acid (FA) oxidation metabolic reprogramming and increased uptake of free FAs, stimulating cancer cell proliferation. Furthermore, inhibition of TGF-β eliminated APOA2-mediated EndoMT and cancer lipid metabolism reprogramming. Notably, HCC with high expression of APOA2 relied on TGF-β to promote cell proliferation and angiogenesis, and pharmacological loss of TGF-β function can reduce angiogenesis and malignant tumor proliferation. These findings reveal an effective cancer therapy concept by inhibition of TGF-β, targeting angiogenesis and lipid metabolism reprogramming.

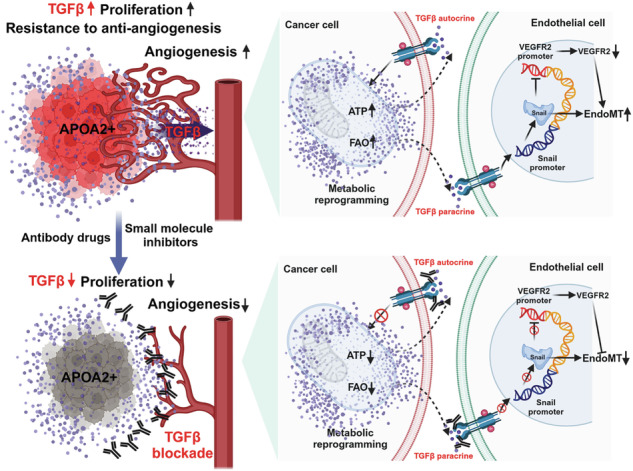

## Introduction

Hepatocellular carcinoma (HCC) is the fourth leading cause of cancer-related mortality globally [1], with more than half of patients diagnosed at an advanced stage, resulting in a poor prognosis [2]. HCC is characterized as a vascular-rich tumor, and antiangiogenic drugs (AADs) have been recommended as the standard treatment for advanced HCC [3].In recent years, there have been approvals of several single drugs, such as lenvatinib in the first and regorafenib and apatinib in the second line for advanced HCC [4]. Current trends indicate that AAD therapy, whether used alone or in combination, will emerge as the primary systemic treatment for HCC in the near future [3, 5]. Currently, the AADs for HCC are tyrosine kinase inhibitors (TKIs) targeting the vascular endothelial growth factor (VEGF) system [6]. In addition to VEGF, other drivers of angiogenesis, such as angiopoietins, placental growth factor, and transforming growth factor beta (TGF-β) [6], can also drive tumor angiogenesis. Tumor endothelial cells (ECs) can maintain angiogenesis through pathways independent of VEGF system, leading to resistance of most HCC to AADs in clinical practice [6–8].

Unlike healthy cells, even in low oxygen and nutrient-deprived microenvironments, cancer cells must efficiently produce energy to support unlimited proliferation, invasion, and metastasis [9]. In addition to glucose metabolism, cancer cells can also rely on lipid metabolism for energy supply [10]. Some studies have shown that highly proliferative cancer cells absorb exogenous lipids and activate endogenous lipid biosynthesis. Through fatty acid (FA) oxidation pathways, they utilize exogenous free FA to generate energy [10]. Lipid metabolism reprogramming in cancer cells contributes to their survival, proliferation, migration, and resistance to AADs [11]. Apolipoprotein A2 (APOA2), the second most abundant apolipoprotein in high-density lipoprotein particles, plays a crucial role in lipid metabolism [12]. Overexpression of APOA2 in transgenic mice increases insulin levels and reduces the hydrolysis of triglycerides in adipose tissue, resulting lipid accumulation and insulin resistance [13]. APOA2 has been considered as a cancer biomarker for early diagnosis of various cancers, but its role in cancer remains unknown [14, 15]. We show that APOA2 endows HCC cells with high proliferation and low apoptosis through lipid metabolic reprogramming, leading to resistance to AADs.

HCC cells exist within a tumor microenvironment (TME) composing various cell types, with ECs notably contributing to tumor angiogenesis [16]. Evidence suggests that ECs undergo phenotypic changes, transitioning into mesenchymal cells via endothelial-to-mesenchymal transition (EndoMT), thus facilitating tumor progression and metastasis [17]. This cellular plasticity, mediated by cytokines such as TGF-β, implicates EndoMT in diverse aspects of tumor progression, including angiogenesis and resistance to cancer treatment [18, 19]. Angiogenesis can be viewed as a part of EndoMT, where EndoMT induced by PDGF correlates with decreased expression of VEGFR2 in ECs, leading to resistance to AAD therapy [7, 18]. TGF-β serves as the primary inducer of EndoMT [20]. Our results show that HCC cells overexpressing APOA2 promote TGF-β secretion, thereby inducing EndoMT and reducing VEGFR2 levels in ECs.

In this study, we uncover that cancer cells with high APOA2 expression oversecrete TGF-β into TME, leading to resistance to AAD therapy. On the one hand, TGF-β acts on ECs, triggering EndoMT and down-regulating VEGFR2 expression, while on the other hand, it affects cancer cells, leading to lipid metabolism reprogramming, ensuring their continued high proliferation and low apoptosis characteristics during anti-angiogenic therapy. TGF-β blockade in an HCC-bearing mouse model reversed this phenomenon.

## Results

### High expression of APOA2 was significantly associated with HCC resistance to AAD therapy

**Fig. 1:**
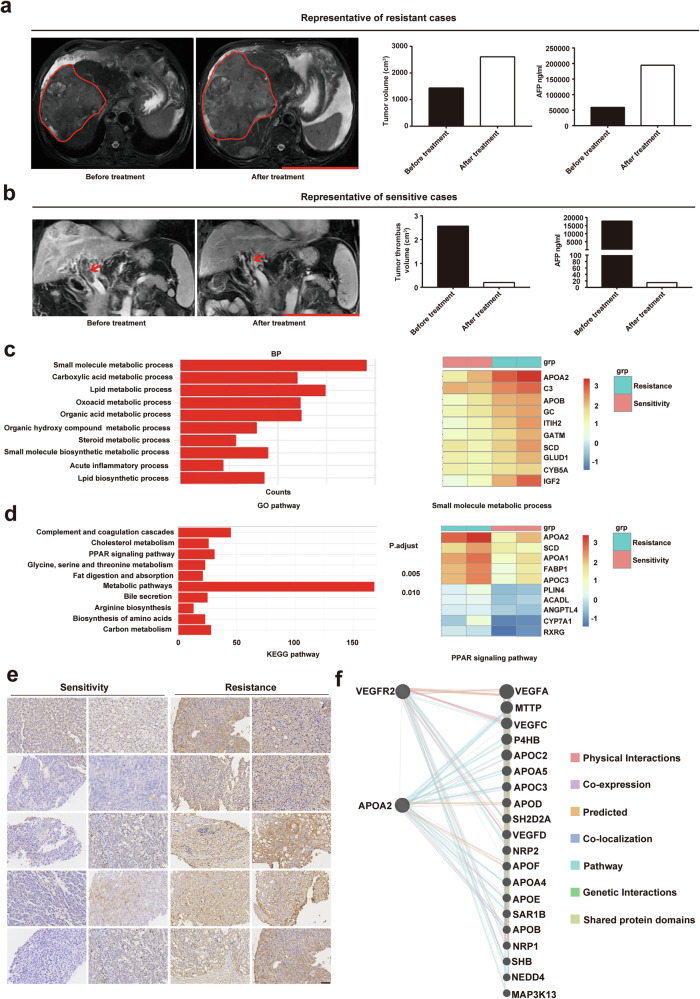
High expression of APOA2 is highly correlated with resistance to AAD therapy in HCC. **a** MRI images, changes in AFP, and quantification of tumor volume in representative HCC-resistant patients before and after apatinib treatment. Scale bars, 10 cm. **b** MRI images, changes in AFP, and quantification of tumor thrombus volume in representative HCC-sensitive patients before and after apatinib treatment. Scale bars, 10 cm. **c** The GO pathway analysis of differential genes was enriched, with APOA2 ranking first in the small molecule metabolic process. **d** The KEGG pathway analysis of differential genes was enriched, with APOA2 ranking first in the PPAR signaling pathway. **e** Intensity of APOA2 IHC staining in 10 paired HCC patient tissues (sensitive vs resistant). Scale bars, 50 µm. **f** Predicted network diagram of the interactions between APOA2 and VEGFR2.

In our previous study [21], we applied for a clinical trial of apatinib monotherapy for patients with advanced HCC, and obtained puncture specimens before apatinib treatment. Through full transcriptome sequencing of these specimens, we identified key signaling pathways and molecules associated with apatinib resistance in HCC patients. We sequenced puncture specimens from 10 apatinib-resistant patients and 10 apatinib-sensitive patients. The MRI images before and after treatment, as well as AFP levels, between representative sensitive and resistant HCC patients were shown in Fig. 1a, b and Supplementary Table 2. After quality inspection, only 2 resistant and 2 sensitive puncture specimens met the full transcriptional sequencing criteria. Our enrichment analysis of GO and KEGG pathways revealed that genes associated with AAD resistance and sensitivity were mainly enriched in metabolite-related pathways, particularly lipid metabolism pathways, consistent with previous studies [11]. We focused on the gene APOA2, which ranks first in relation to lipid metabolism, to verify its correlation with AAD resistance (Figs.1c, d). Immunohistochemistry (IHC) of APOA2 in 10 apatinib-resistant and 10 apatinib-sensitive specimens showed significantly higher levels of APOA2 in resistant specimens compared to sensitive specimens (Fig.1e). Using the GeneMANIA website, we explored the correlation between the expression of APOA2 and VEGFR2, a key AAD target, and found a correlation between APOA2 and VEGFR2 co-expression (Fig.1f). These results confirmed that elevated APOA2 expression is positively associated with HCC resistance to AAD therapy.

### The tumor tissue of certain acquired AAD-resistant HCC-bearing mouse model exhibited elevated expression of APOA2 and decreased expression of VEGFR2

**Fig. 2:**
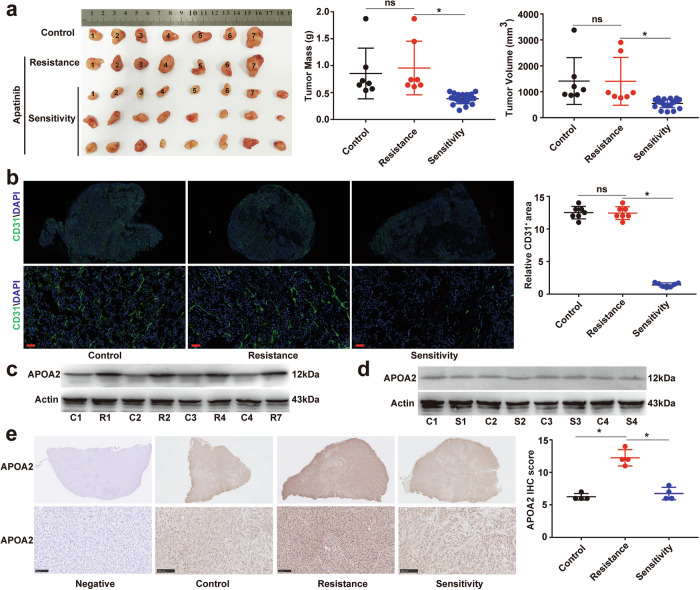
APOA2 is responsible for acquired resistance to AAD in HCC-bearing mouse model. After the evaluation of acquired drug resistance, the HCC-bearing mice were divided into 3 groups: (C)control (*n* = 7), (R)resistant (*n* = 7), and (S)sensitive (*n* = 24). **a** Quantification of subcutaneous tumor mass and volume in the three groups. Numbers 1–7 represent control group, 1–7 for resistance group, and 1–7 for sensitivity group. *p < 0.05, ns, not significant. **b**. Representative images of CD31 immunofluorescence staining of tumor tissue. The upper part of the image is a comprehensive view of CD31 staining of the entire tumor tissue section, while the bottom part shows CD31 staining under magnification. CD31^+^ microvessel signals were randomly quantified from 12 fields (*n* = 7 mice in each group), Scale bars, 50 µm. *p < 0.05, ns, not significant. **c** and **d**. Western blotting of APOA2, VEGFR2, and P-VEGFR2 in tumor tissue (*n* = 4). **e** Representative images of APOA2 IHC staining intensity of tumor tissue, Scale bars, 100 µm. The upper part of the image is a comprehensive view of APOA2 staining of the entire tumor tissue section, while the bottom part shows APOA2 staining under magnification. Scale bars, 100 µm. ^*^p < 0.05, ns, not significant.

Due to AAD’s targeting the vascular microenvironment of HCC, there is currently no appropriate in vitro research model. To further explore the correlation between APOA2 and AAD resistance, we established an HCC-bearing mouse model by subcutaneously injecting MHCC-97H cells into the lateral forearm of male BALB/c (nu/nu) mice. After tumor formation, mice were given apatinib daily, and those exhibiting acquired apatinib resistance were identified, as shown in Fig. 2a. Compared with control group, 7 apatinib-resistant mice were identified. Using CD31 immunofluorescence staining of total tumor tissue, we demonstrated that angiogenesis in resistant tumors remained unaffected by apatinib therapy (Fig. 2b). Additionally, we analyzed the protein levels of APOA2 and VEGFR2 in resistant and sensitive tumor masses and found that APOA2 protein levels increased and VEGFR2 protein levels decreased in resistant tumor masses, while the protein levels of APOA2 and VEGFR2 did not change in sensitive tumor masses (Fig. 2c, d). Then we detected the APOA2 level of total tumor tissue using immunohistochemical method and confirmed that the APOA2 level in resistant tumor masses was indeed higher than in sensitive tumor mass (Fig. 2e). These results suggest that APOA2 may contribute to resistance to AAD therapy in some HCC patients.

### Overexpression of APOA2 led to HCC resistance to AAD in vivo

**Fig. 3:**
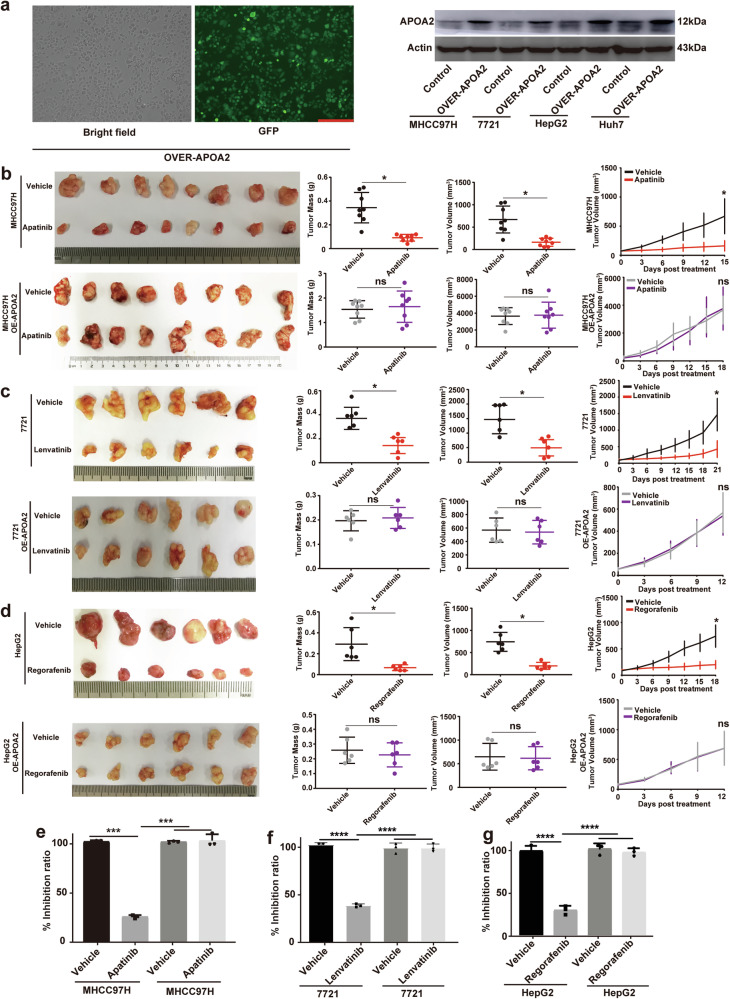
APOA2 overexpression leads to resistance to anti-angiogenic therapy in HCC. **a** Representative images of HCC cell lines overexpressing APOA2. Scale bars, 100 µm. Western blotting verified the protein levels of APOA2, VEGFR2, and P-VEGFR2 in 4 cell lines. **b**–**d** Subcutaneous tumor, tumor volume and tumor mass quantification, along with tumor growth curve of each group (*n* = 6). **e**–**g** Characterization of tumor inhibition rate in each group. ^***^p < 0.001, ^****^p < 0.0001, ns not significant.

To explore whether regulation of APOA2 can induce AAD resistance in HCC, we used lentiviral infection to overexpress APOA2 in 4 HCC cell lines (MHCC97H, SMMC-7721, Huh7, and HepG2). Western blotting showed that APOA2 overexpression decreased VEGFR2 protein levels in cell lines (Fig. 3a). We established HCC-bearing mouse models using three HCC cell lines overexpressing with or without APOA2, and then the tumor-bearing mice were treated with three commonly used AAD (apatinib, regorafenib, and lenvatinib). Analysis of tumor volume, mass, and tumor inhibition rate demonstrated that APOA2 overexpression conferred resistance to all three AAD drugs in HCC (Fig. 3c–g). These findings confirmed that APOA2 overexpression led to resistance to anti-angiogenic therapy in HCC.

### APOA2 promoted HCC cell proliferation and decreased apoptosis in vitro and in vivo

**Fig. 4:**
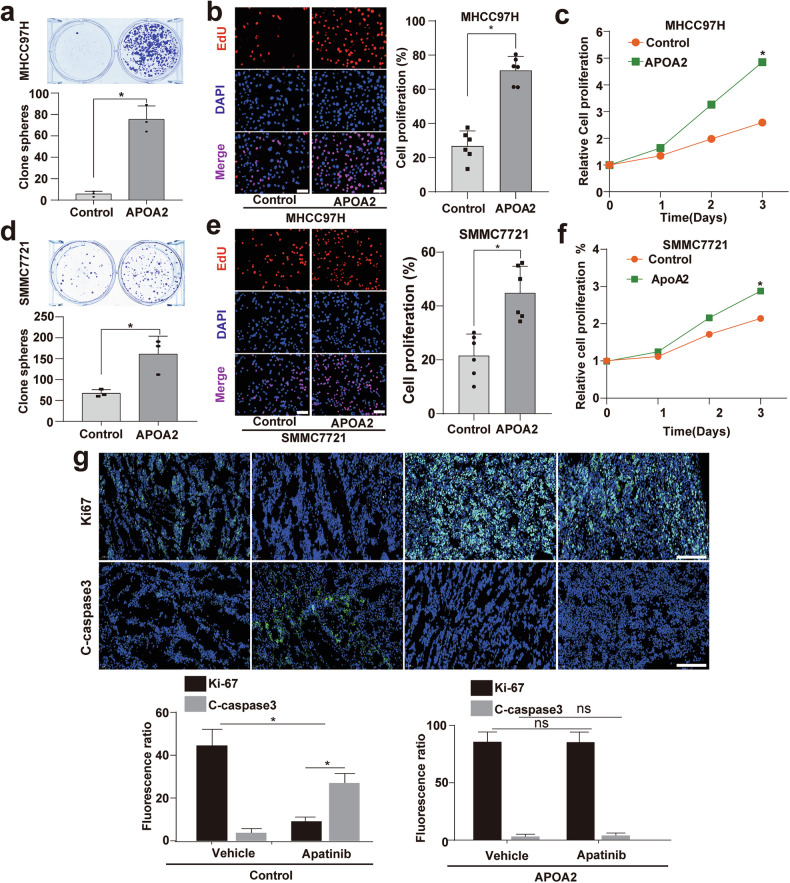
APOA2 overexpression confers high proliferation and low apoptosis characteristics on HCC post-AAD treatment. **a**–**f** The experiment comprised two groups: control (Control) and APOA2 overexpression (APOA2). **a**, **d** Colony formation assay conducted on MHCC97H and SMMC7721 cells (*n* = 3), **b**, **e** Representative images from EdU assay performed on MHCC97H and SMMC7721 cells, with EdU signals quantified from 12 fields (*n* = 6), Scale bars, 50 µm. **c**, **f** CCK8 assay results of MHCC97H and SMMC7721 cells. **g** The experiment comprised 4 groups: control group receiving solvent and apatinib (Control + Vehicle vs Control + Apatinib), and the APOA2 overexpression group receiving solvent and apatinib (APOA2+Vehicle vs APOA2+Apatinib). Representative images of Ki67 and cleaved-caspase 3 immunofluorescence staining of tumor tissue, with Ki67+ and cleaved-caspase 3+ signals quantified from 12 fields (*n* = 6). Scale bars, 100 µm. ^*^p < 0.05, ns not significant.

To explore why APOA2 causes AAD resistance, we examined its effect on the HCC cells. Colony formation assay (Fig. 4a, d), EdU (5-ethynyl-2’ -deoxyuridine) assay (Fig. 4b, e) and CCK8 (Cell Counting Kit-8) experiments (Fig. 4c, f) showed that APOA2 promoted the proliferation of HCC cells. Since AAD drugs such as apatinib are insoluble in water, achieving stable drug concentrations in vitro is challenging. Hence, we evaluated the effect of apatinib on HCC cells overexpressing APOA2 in vivo. Immunofluorescence staining with Ki67 and cleaved-caspase3 showed that APOA2 overexpression promoted proliferation and reduced apoptosis in HCC cells during apatinib treatment (Fig. 4g). Overall, these results suggest that overexpression of APOA2 confers HCC cells with characteristics of high proliferation and low apoptosis, on even after AAD treatment.

### The conditional culture medium of HCC cells overexpressing APOA2 promoted proliferation, migration, and tubular formation in umbilical vein endothelial cells (HUVECs)

**Fig. 5:**
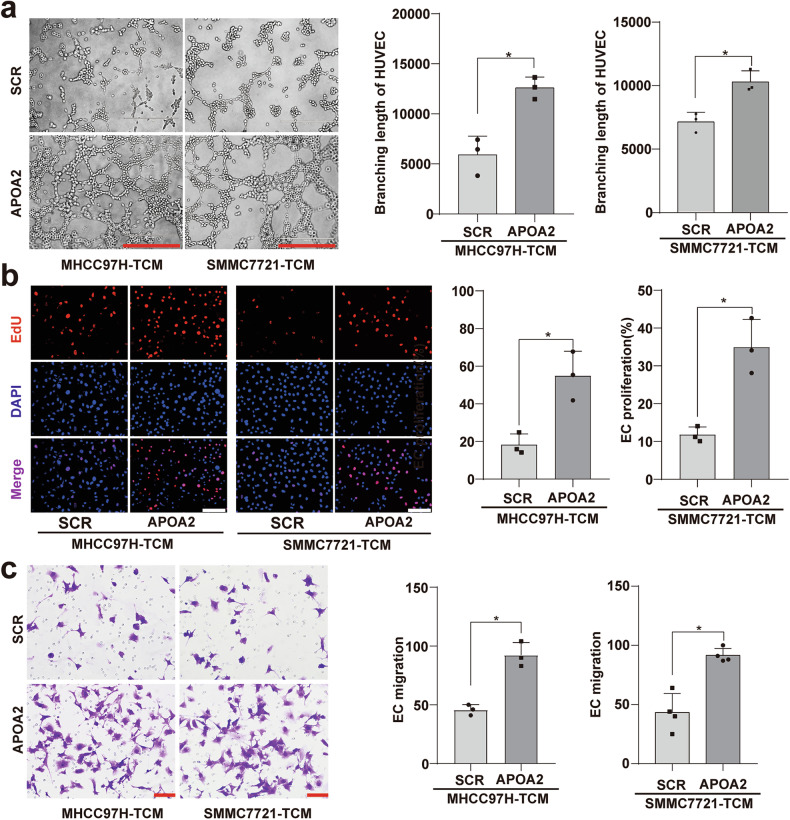
The conditional medium from HCC cells overexpressing APOA2 on HUVEC proliferation, migration, and tubular formation. **a** Representative bright-field images of tubular formation in HUVECs cultured with conditioned media from MHCC97H and SMCC7721 (overexpressing APOA2 or not). Scale bar, 400 µm. **b** Representative images of EdU assay conducted on HUVECs cultured with conditioned media from MHCC97H and SMCC7721 (overexpressing APOA2 or not), with EdU signals quantified from 12 fields (*n* = 6). Scale bars, 100 µm. **c** Representative images of migration of HUVECs cultured with conditioned media from MHCC97H and SMCC7721 (overexpressing APOA2 or not), Scale bars, 100 µm. ^*^p < 0.05, ns not significant.

We further explored the effect of HCC overexpression of APOA2 on the vascular microenvironment. In order to simulate the in vivo effect of HCC overexpression of APOA2 on the vascular microenvironment, we observed the effect of conditioned medium from HCC cells overexpression APOA2 on human HUVECs in vitro. HUVECs were cultured with conditioned media from MHCC97H and SMMC-7721 cells (either overexpressing APOA2 or not). The results revealed that overexpressing APOA2 promoted HUVECs proliferation (Fig. 5b), migration (Fig. 5c), and tubule formation (Fig. 5a). However, as shown in Fig. 3a, HCC cells overexpressing APOA2 exhibited decreased the level of VEGFR2, suggesting that these cells produced pro-angiogenic factors other than VEGFA in the vascular microenvironment.

### Overexpression of APOA2 activated the TGF-β signaling pathway and increased TGF-β secretion in HCC

**Fig. 6:**
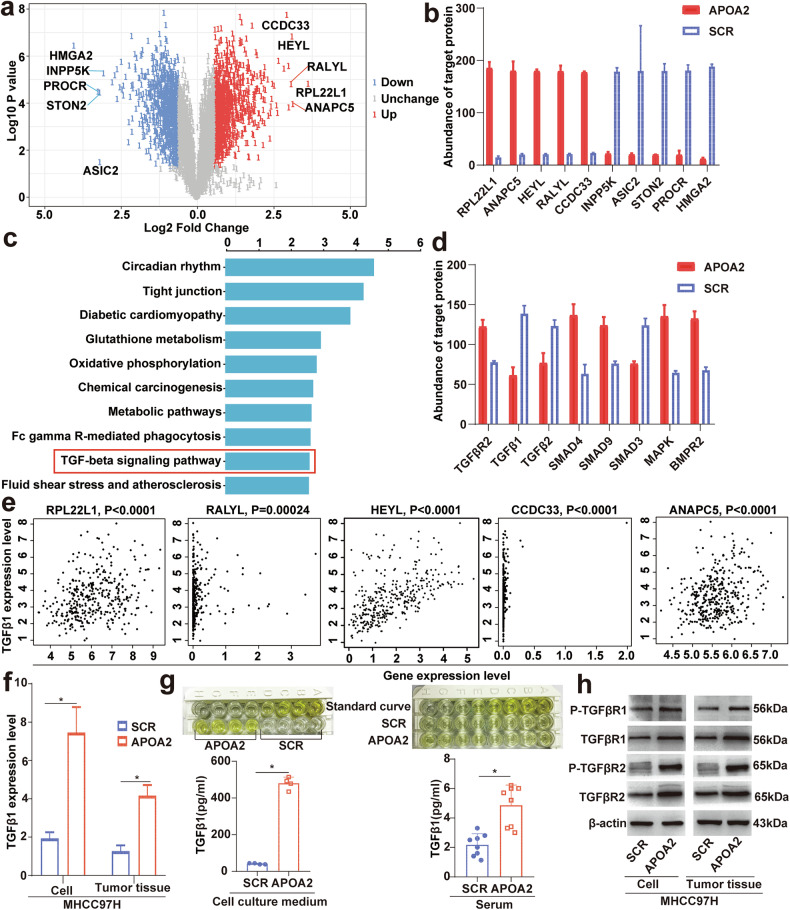
APOA2 overexpression increases TGF-β secretion and activates the TGF-β signaling pathway in HCC. **a** Volcano plot of differentially expressed proteins between the APOA2 overexpression group and the control group, with a fold change ≥ 1.5 and adjusted *p* < 0.01 (*n* = 3). Gray denotes no significant difference, blue indicates low expression, and red indicates high expression. The volcano plot labels the five highest expressed proteins and five lowest expressed proteins. **b** Histogram of 5 highest expressed proteins and 5 lowest expressed proteins (*n* = 3). **c** Enrichment analysis of KEGG pathways for differentially expressed proteins between the APOA2 overexpression group and the control group. **d** Histogram showing levels of TGF-β ligand, receptor, and signaling pathway-related protein in proteomics (n = 3). **e** Pearson correlation analysis of five highly expressed proteins and TGF-β in HCC. **f** Relative mRNA levels of TGF-β in HCC cells and tumor tissues with or without overexpressing APOA2 analyzed by real-time quantitative PCR (*n* = 6). **g** ELISA detection of TGF-β in HCC cell (with or without overexpressing APOA2) medium and serum of HCC-bearing mice (with or without overexpressing APOA2). **h** Western blotting detecting the protein levels of TGF-βR1, TGF-βR2, P-TGFβR1 and P-TGFβR2 in HCC cells and tumor tissues with or without overexpressing APOA2. ^*^p < 0.05, ns, not significant.

To investigate the pro-angiogenic factors produced in the vascular microenvironment of HCC overexpressing APOA2, we performed quantitative proteomics on MHCC97H cells with or without APOA2 overexpressing (3 vs 3). A total of 4992 proteins were detected, among which 1995 proteins showed significant differences. The volcano plot depicting the protein landscape is shown in Fig. 6a. Enrichment of the KEGG signaling pathway revealed activation of the TGF-β signaling pathway associated with pro-angiogenesis factors (Fig. 6c). Among the significant different proteins, the levels of TGF-β receptor and its downstream related proteins were found to increase, while ligand levels decreased (Fig. 6d), indicating that ligands may be secreted into TME to regulate angiogenesis. Based on the volcano plot, we selected the 5 highest expressed proteins and 5 lowest expressed proteins (RPL22L1, ANAPC5, HEYL, RALYL, CCDC33, INPP5K, ASIC2, STON2, PROCR, and HMGA2) (Fig. 6b). Among them, RPL22L1[22], HEYL[23], RALYL[24], INPP5K, ASIC2 [25], and HMGA2 [26] were related to the activation of the TGF signaling pathway. Subsequently, we verified that the five highest expressed proteins (RPL22L1, ANAPC5, HEYL, RALYL and CCDC33) were positively correlated with TGF-β expression using the GEPIA website (Pearson correlation, Fig. 6e). RT-PCR confirmed that TGF-β expression was upregulated in HCC cells and tumor tissues with APOA2 overexpression (Fig. 6f). ELISA assays confirmed increased TGF-β secretion into TME due to APOA2 overexpression in HCC (Fig. 6g). Western blotting detected the levels of phosphorylation of TGF-β receptor 1 (P-TGFβR1) and receptor 2 (P-TGFβR2) in HCC cells and tumor tissues, confirming that overexpression of APOA2 activated the TGF-β signaling pathway. Collectively, these results suggest that angiogenesis in HCC with high APOA2 expression is more dependent on TGF-β signaling than VEGF signaling.

### Overexpression of APOA2 induced lipid metabolic reprogramming through TGF-β signaling in HCC

**Fig. 7:**
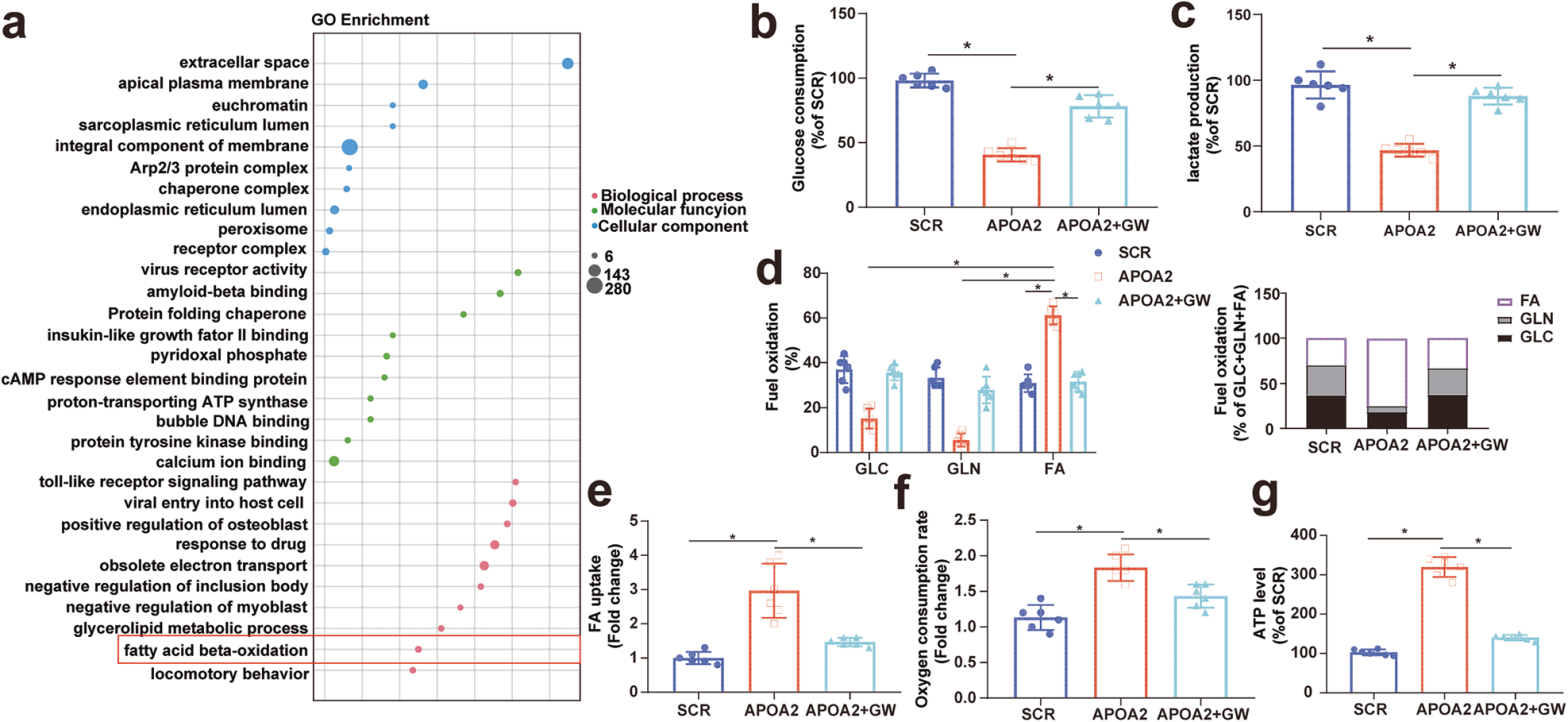
APOA2 overexpression of induces lipid metabolic reprogramming through TGF-β signaling in HCC. **a** Enrichment analysis of GO pathways for differentially expressed proteins between APOA2 overexpression and the control group, with a fold change ≥ 1.5 and adjusted *p*-value < 0.01. **b** Glucose consumption in MHCC97H cells in the designated group (*n* = 6). **c** Lactate production in MHCC97H cells in the designated group (*n* = 6). **d** Mito Fuel Flex test revealed dependence on the oxidation of glucose, glutamine, or FA in MHCC97H cells in the designated group (*n* = 6). **e** BODIPY-FA uptake in MHCC97H cells in the designated group. **f** β-oxidation assay in MHCC97H cells in the designated group (*n* = 6). **g** Intracellular ATP levels in MHCC97H cells in the designated group (*n* = 6). ^*^p < 0.05, ns not significant.

Cancer lipid metabolism contributes to AAD resistance [11]. APOA2 influences lipid metabolism [27] and enhances FA uptake and β-oxidation in cancer cells upon TGF-β stimulation [28]. Figure 4 suggested that APOA2 overexpression led to high proliferation and low apoptosis in HCC, while Fig. 6 confirmed the activation of the TGF-β signaling pathway by APOA2. Therefore, it is plausible that APOA2 promotes lipid reprogramming in HCC through TGF-β signaling. KEGG and GO analyses showed that APOA2 involvement in the processes of glutathione metabolism, oxidative phosphorylation, metabolic pathways, and fatty acid β-oxidation (FAO) (Figs. 6c and 7a). To investigate APOA2-mediated lipid reprogramming in HCC via TGF-β signaling, we categorized the experiment into 3 groups: blank control (SCR), APOA2 overexpression (APOA2), and APOA2 overexpression +TGF-β inhibitor treatment (GW). Analysis of the glycolytic levels in MHCC97H cells revealed that decreased glucose consumption and lactate production upon APOA2 overexpression, which was reversed by TGF-β inhibitors (Fig. 7b, c). Seahorse mito fuel assay illustrated a heightened dependency on FAO rather than glucose or glutamine in MHCC97H cells upon APOA2 overexpression, which was reversed by TGF-β inhibitors (Fig. 7d). Moreover, fluorescent BODIPY FA probe demonstrated increased FAs intake upon APOA2 overexpression, which was mitigated by TGF-β inhibitors in MHCC97H cells (Fig. 7e). Furthermore, measurement of the palmitate (PA)-based oxygen consumption rate (POCR) revealed enhanced POCR and ATP production upon APOA2 overexpression, which was attenuated by TGF-β inhibitors (Fig. 7f, g). These findings substantiate that APOA2 upregulates FAs uptake and activates the FAO pathway, inducing lipid metabolic reprogramming via TGF-β signaling in HCC.

### The angiogenesis of HCC overexpressing APOA2 depended on TGF-β signaling rather than the VEGFA-VEGFR2 signaling pathway, as TGF-β reduced the expression of VEGFR2 in ECs and promoted EndoMT

**Fig. 8:**
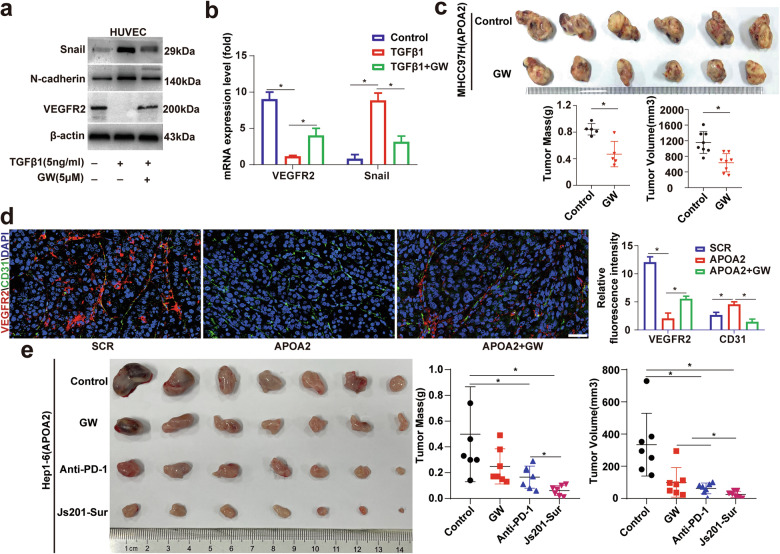
Angiogenesis in HCC with high APOA2 expression is TGF-β dependent sensitive to TGF-β inhibitors, and more sensitive to PD-1/TGF-β dual antibodies. **a** Western blotting detecting the protein levels of Snail, N-cadherin, VEGFR2 in HUVEC in the designated group. **b** Relative mRNA level of Snail in HUVEC in the designated group was analyzed by real-time quantitative PCR (*n* = 6). **c** Subcutaneous tumor, tumor volume, and tumor mass quantification of each group (*n* = 6). **d** Representative images of CD31 and VEGFR2 immunofluorescence staining of tumor tissue, with CD31+ andVEGFR2+ signals quantified from 12 fields (*n* = 6). Scale bars, 40 µm. **e** Assessment of subcutaneous tumor growth, tumor volume, and tumor mass for each group treated with GW, GW788388 (TGF-β inhibitors), anti-PD-1 (anti-mouse PD-1 antibody), and JS201-Sur (mouse PD-1/TGF-β dual antibody) (*n* = 6). ^*^p < 0.05, ns, not significant.

Tumor angiogenesis is considered a partial EndoMT [29], with TGF-β recognized as a major inducer of EndoMT [18, 29], and ECs exposed to TGF-β lose VEGFR2 signaling [30]. Figure 6 showed that overexpression of APOA2 increased TGF-β secretion in HCC. We further confirmed that APOA2 acted on ECs and HCC cells through TGF-β, resulting in decreased expression of VEGFR2 (AAD target), promoting EndoMT in ECs, and further promoting angiogenesis (Fig. 8a, b, d). Figure 3 illustrated that overexpression of APOA2 conferred resistance to AAD drugs on HCC, and Fig. 6 suggested that angiogenesis in HCC with high APOA2 expression was more dependent on TGF-β signaling than VEGF signaling. Our data confirmed that HCC overexpressing APOA2 was sensitive to TGF-β inhibitors (Fig. 8c). TGF-β can also affect the tumor immune microenvironment and promote the immunosuppression and immune escape of tumor cells [31, 32]. JS201, the first PD-1/TGF-β dual antibody developed by Junshi Biosciences, has been approved for clinical trials. JS201 blocks the PD-1/PD-L1 pathway and neutralizes TGF-β in TME. JS201-Sur, a mouse PD-1/TGF-β dual antibody from Junshi Biosciences, was investigated for its effect on HCC after overexpressing APOA2 in the murine HCC cell line Hep1-6. The results showed that JS201-Sur had a better anti-cancer effect on HCC overexpressing APOA2 than TGF-β inhibitor and PD-1 antibody alone. Taken together, these results suggest that angiogenesis in HCC with high APOA2 expression is dependent on TGF-β signaling rather than VEGFA-VEGFR2 signaling. These HCC patients are resistant to AAD but sensitive to TGF-β inhibitors and more sensitive to PD-1/TGF-β dual antibodies. We identify an APOA2/TGF-β axis that controls EndoMT and VEGFR-2 down-expression in ECs and lipid metabolism reprogramming in cancer cells.

## Discussion

Angiogenesis presents a hallmark of HCC development and progression [33], while drug resistance poses a significant challenge to achieving high efficiency with AADs in HCC patients [34]. AAD treatment often leads to intrinsic and acquired drug resistance in HCC patients, limiting its therapeutic efficacy and providing only marginal survival benefits to most individuals [35]. Despite these known clinical facts, the molecular mechanisms underlying AAD resistance remain largely unknown [36]. Most studies have focused on the compensatory mechanism, namely the drug-induced conversion of the angiogenic signaling pathway. Current anti-angiogenic therapies primarily target the VEGFA/VEGFR-2 signaling pathway in HCC treatment. Solely targeting this pathway may inadequately eradicate and inhibit tumor-associated ECs [7, 37]. We propose vascular detransformation as a novel anti-angiogenic strategy for certain HCC patients. Our study showed that EndoMT eliminated VEGFR2 expression in certain HCC-associated ECs populations. These ECs may undergo mesenchymal transition and gene reprogramming, bypassing the angiogenic pathway to support survival, proliferation, and migration within TME, evading cytotoxic effects induced by conventional anti-angiogenic therapies targeting VEGFA/VEGFR-2 signaling. Similarly, our findings indicate strong resistance of certain HCC-derived ECs to VEGF/VEGFR-2 therapy, challenging the VEGFA/VEGFR2 signaling pathway as the primary target for anti-angiogenic therapy.

ECs within the HCC microenvironment exhibit remarkable plasticity, undergoing EndoMT characterized by enhanced proliferation and migration, which contributes to vascular abnormalities and treatment resistance. Despite undergoing EndoMT, ECs maintain critical endothelial functions. Importantly, we showed that APOA2 induces low VEGFR2 expression in ECs via TGF-β. The downregulation of VEGFR2 expression suggests that the transformed ECs develop mechanisms independent of VEGFA to sustain growth and survival in HCC, rendering them resistant to anti-VEGFA treatment. Consequently, the mesenchymal phenotype acquired by ECs in HCC leads to overgrowth of these drug-resistant transformed ECs in TME. Hence, precisely targeting ECs transformation holds promise as a cancer treatment strategy. Despite advancements, the regulatory mechanisms of EndoMT remain largely elusive. EC plasticity is mediated by various pathways, including TGF-β, bone morphogenetic protein, HGF/c-Met and Notch pathways [38, 39]. Recent studies have highlighted the essential role of snail in hypoxic, TGF-β treatment-induced EC plasticity [40, 41]. Here, we identified snail as a key downstream regulator of EndoMT in HCC, demonstrating that TGF-β induced snail expression mediates EndoMT and downregulates VEGFR2 expression.

The growth of HCC depends on angiogenesis, but recent studies indicate that AADs anti-angiogenesis effect does not inhibit tumor growth near adipose tissue and in fatty liver. In models involving adipose tissue and fatty liver implantation tumors, anti-VEGFA therapy showed significant antiangiogenic effects, with AAD-induced hypoxia driving the transition to lipid metabolic reprogramming in tumor cells. Key molecular components involved in FAO, including AMPK, were significantly upregulated, suggesting that the FAO pathway activation in AAD-treated tumors. Moreover, hypoxia significantly increases FAs uptake and transport in malignant cells [11]. Notably, our study suggests that APOA2 promotes FAs uptake and activates the FAO pathway in HCC via TGF-β. Our research elucidates how APOA2 promotes lipid metabolism in HCC and contributes to AAD resistance. We propose that complete eradication of the HCC vascular system may necessitate simultaneous targeting of EndoMT and lipid metabolic reprogramming. Importantly, our proteomic analysis and subsequent experiments identified TGF-β as a key regulator of EndoMT and lipid reprogramming regulated by APOA2, which decreased VEGFR2 expression in ECs and promoted lipid metabolism in HCC. Inhibiting TGF-β in HCC patients with high APOA2 expression could be an effective approach. These findings provide a good therapeutic paradigm for effectively treating HCC by targeting both angiogenesis and lipid metabolism.

Systemic treatment of advanced HCC has relied on TKIs until the advent of immune checkpoint inhibitors (ICIs), offering new therapeutic avenues. However, single-drug resistance and suboptimal efficacy necessitate novel strategies, such as combining ICIs with AADs or multiple ICIs [42]. The combination of anti-angiogenesis therapy and ICIs in HCC addresses some aspects of AAD resistance [43]. Within TME, the PD-L1/PD-1 pathway and TGF-β signaling are pivotal in tumor immune escape [32]. TGF-β upregulates PD-L1 on antigen-presenting cells, release it into TME, where it inhibits cytotoxic T lymphocytes directly or prompts them to release inhibitory soluble molecules. Therefore, targeting and neutralizing TGF-β in TME alongside PD-1/PD-L1 inhibition could restore T cell activity and enhance the immune response, offering a promising therapeutic approach [31]. Our data confirm that in HCC with high APOA2 expression, angiogenesis depends on the TGF-β signaling pathway rather than the VEGFA signaling pathway, rendering it sensitive to TGF-β inhibitors and but insensitive to AAD therapy. However, the therapeutic efficacy of TGF-β blockade may be constrained by several factors. The immunosuppressive tumor microenvironment in HCC is multifactorial, and compensatory immune evasion mechanisms may limit the durability of response to TGF-β inhibition alone. Furthermore, tumor heterogeneity implies that not all APOA2-high HCCs will respond equally [44, 45]. Elevated TGF-β levels in the TME of APOA2-expressing HCC likely create an immunosuppressive milieu. Our results confirm that PD-1/TGF-β dual antibody exhibits superior anti-cancer effect on HCC with high APOA2 expression compared to single PD-1 or TGF-β inhibition. Currently undergoing clinical trials, PD-1/TGF-β dual antibody holds promise for treating HCC with high APOA2 expression. While PD-1/TGF-β dual antibody represents a promising strategy to overcome these limitations, future studies should identify robust biomarkers to precisely stratify patients who would benefit most from this combinatorial approach. Unfortunately, attempts to establish stable cell lines by lentiviral transfection with APOA2 knock-out or knock-down in HCC cells have been unsuccessful, possibly due to cell death resulting from APOA2 depletion. Therefore, we were unable to observe changes in metabolism and vascular microenvironment in HCC with little or no APOA2 expression.

## Conclusion

This study demonstrated that APOA2 is a key driver of antiangiogenic therapy resistance in HCC. We delineate a mechanism whereby tumor-derived APOA2 orchestrates a dual-pronged strategy for evasion: it stimulates TGF-β secretion, which in turn induces EndoMT and downregulates VEGFR2 in endothelial cells, thereby rendering them insensitive to VEGF-targeted therapies. Concurrently, TGF-β reprograms cancer cell lipid metabolism, enhancing fatty acid uptake and oxidation to fuel proliferation and suppress apoptosis under AAD pressure. This APOA2/TGF-β axis thus coordinately sustains both the vascular niche and tumor cell fitness. The reversal of resistance by TGF-β pathway blockade and the superior efficacy of a PD-1/TGF-β dual-specific antibody underscore the therapeutic vulnerability created by this pathway. Our study not only elucidates a previously unrecognized mechanistic link between a lipid metabolism regulator and angiocrine signaling in therapy resistance but also nominates combined targeting of TGF-β and immune checkpoints as a promising strategy for a subset of HCC patients with high APOA2 expression.

## Materials and methods

### HCC patients and tissue specimens

Patient tissue specimens were sourced from follow-up data of participants in our clinical trials (apatinib study in advanced HCC). These trials encompassed clinically identified HCC cases categorized as either resistant or sensitive to apatinib. Data, including patients’ AFP levels and imaging results, were extracted from the record system of Tianjin Medical University Cancer Institute and Hospital, spanning from January 2016 to December 2020. Further information on the clinical trial can be found at https://clinicaltrials.gov/ (identifier NCT03046979).

### Full transcriptome sequencing of HCC tissues

The sequencing methodology follows our prior work (Chen et al., 2020), while gene enrichment analysis utilized ClusterProfiler with an FDR ≤ 0.05 cutoff. Raw sequenced data are available from the corresponding authors upon request. Interaction potential between APOA2 and VEGFR2 was assessed using the GeneMANIA website (https://genemania.org/).

### Immunohistochemistry of APOA2

Immunohistochemical staining protocols were consistent with our previous investigation (Zhang et al., 2021). Primary APOA2 antibody (Proteintech, 16845-1-AP, CHN) was diluted at 1:50, while mouse/rabbit linker (Santa Cruz, sc2357, USA) was diluted at 1:100. Each immunostaining run incorporated suitable positive and negative controls.

### Cell lines and transfections

SMCC-7721, HepG2, and HUVEC cells were sourced from the American Type Culture Collection (ATCC; Manassas, VA, USA). Huh7 cells were procured from the Health Science Research Resources Bank (Shanghai, China). MHCC97H and Hep1-6 cells were generously provided by the Liver Cancer Institute of Zhongshan Hospital, Fudan University. These cell lines were maintained in complete DMEM medium supplemented with 10% fetal bovine serum (FBS; PAN-Seratech) and 1% penicillin-streptomycin solution (PS; HyClone) under standard culture conditions (37 °C, 5% CO2). Lentiviruses for APOA2 overexpression were generated by Shanghai Ji Kai Gene Chemical Technology Co., Ltd., and verified by DNA sequencing. MHCC-97H, SMMC-7721, HepG2, and Huh7 cells were stably infected with the APOA2 overexpression lentivirus and selected with puromycin according to the manufacturer’s instructions.

### Establishment of the HCC-bearing mouse model and administration of GW788388, AADs, anti-mouse PD-1, and Js201-Sur

A human HCC cell suspension (1 × 10^7^ cells) or human HCC cells transfected with APOA2 overexpression lentivirus (1 × 10^7^ cells) in 100 μL PBS was subcutaneously injected into the lateral forearm of 4-week-old male BALB/c nude mice. Similarly, Hep1-6 cells (1 × 10^7^ cells) or Hep1-6 cells overexpressing APOA2 lentivirus (1 × 10^7^ cells) were subcutaneously injected into 4-week-old male C57BL/6 mice. Mice were housed in groups of six per cage at the Experimental Animal Center of Tianjin Medical University Cancer Institute and Hospital under specific pathogen-free conditions. After tumor establishment, mice were randomly assigned to different treatment groups and administered AADs (apatinib, regorafenib, and lenvatinib), TGF-β inhibitor (GW788388), anti-mouse PD-1, or Js201-Sur via oral gavage or intraperitoneal injection. The doses were as follows: apatinib: 200 mg/kg/day (Jiangsu Hengrui Company, China), regorafenib: 100 mg/kg/day (Selleck, S1178), lenvatinib: 30 mg/kg/day (Selleck, S1164), GW788388: 10 mg/kg/day (Selleck, S2750), anti-mouse PD-1: 10 mg/kg/half a week (Selleck, A2122), Js201-Sur: 200 mg/kg/half a week (Junshi Biosciences, China). The amount of anti-PD-1 antibody in the PD-1/TGF-β dual antibody was matched to that in the anti-mouse PD-1 antibody alone. After the experimental period, mice were euthanized, and tumor tissues and blood samples were collected. A portion of the tumor tissues was fixed with 4% paraformaldehyde overnight, followed by embedding in paraffin, while serum samples were stored at −80 °C. Tumor volume and growth curves were calculated according to established methods (Zhang et al.^21^), and the tumor growth inhibition value was determined using the formula: (treatment group tumor weight/control group tumor weight) × 100%.

### Immunofluorescence staining and imaging

Immunohistochemical staining procedures followed those outlined in our previous study (Zhang et al.^21^). Primary antibodies used included CD31 (1:100, Abcam, ab222783, USA), Ki67 (0.5 µg/ml, Abcam, ab15580, USA), Cleaved-caspase3 (1:400, Cell Signaling Technology, # 9664S, USA), and VEGFR2 (1:50, Abcam, ab307623, USA). Secondary antibodies used were goat anti-rabbit IgG H&L (1:500 and 1:1000, Abcam, ab150077 and ab6708, USA). Imaging was conducted using a universal inverted fluorescence microscope (Leica DMI6000B, USA), and ImageJ software was employed for image analysis and quantification.

### Western blotting

Western blotting was conducted using primary antibodies against APOA2 (Abcam, ab185128, USA), P-VEGFR2 (Abcam, ab5473, USA), VEGFR2 (Abcam, ab39256, USA), TGF-βR1 (Abcam, ab235578, USA), TGF-βR2 (Abcam, ab259360, USA), P-TGFβR1 (Abcam, ab112095, USA), P-TGFβR2 (Abcam, ab111564, USA), β-actin (Abcam, ab179467, USA), Snail (Abcam, ab216347, USA), and N-cadherin (Abcam, ab245117, USA). Mouse/rabbit secondary antibodies were obtained from Cell Signaling Technology (#7076, #7074, USA). Protein levels were quantified using Gel-Pro Analyzer Version 4.0

### Cell proliferation assay

Cells were seeded in a 96-well plate at an initial concentration of 2 × 10^3^ cells/well. Each group was set up with five replicates. Cell counting kit-8 (Dojindo Laboratories, Kumamoto, Japan) at 10 μL/well was added, and the cells were cultured at 37 °C with 5% CO2 for 4 h. Optical density (OD) values were measured at a wavelength of 450 nm using an enzyme-labeled instrument. The cell proliferation curve was generated after continuous detection for 4 days.

### Colony formation

Each experimental group was seeded with 700 cells per well in 6-well culture plates. Culturing was continued until most single clones reached a cell count exceeding 50. The cells were then washed once with PBS and fixed with 1 mL of 4% paraformaldehyde per well for 30–60 min. Subsequently, 1 mL of crystal blue dye was added to each well and left for 10–20 min before washing the cells with PBS several times. After drying, images were captured using a digital camera.

### EdU (5-ethynyl-2-deoxyuridine) assay

The EdU solution was diluted with complete cell culture medium to prepare a 2× EdU working solution with a final concentration of 50 μM. Each well received 500 μL of the EdU working solution mixed with 500 μL of culture medium (1:1 ratio). After thorough mixing, the cells were incubated with the EdU solution in the incubator for 6–7 h. Following this, the cells were washed three times with PBS, and 1 mL of 4% paraformaldehyde was added to each well and incubated at room temperature for 15 min. After another three washes with PBS, 1 mL of permeabilization solution (0.3% TritonX-100 PBS) was added to each well and incubated at room temperature for 15 min. Subsequently, 0.2 mL of Click reaction mixture was added to each well, and the culture plate was gently shaken to ensure even coverage of the cells. The reaction was then carried out away from light for 1–2 h. Finally, 1 mL of 1× DAPI solution was added to each well and incubated at room temperature for approximately 10 min, avoiding exposure to light. Images were captured using a universal inverted fluorescence microscope (Leica DMI6000B, USA), and ImageJ software was employed for image analysis and quantification.

### Tube formation assay

HUVEC cells from the 3rd to 5th generations were starved for 24 h. The matrix gel was prepared by diluting it to a concentration of 10 mg/mL with culture medium. This gel was added to a 24-well plate and transferred to the cell incubator for incubation for 30 min to form the basement membrane. A single-cell suspension containing 400,000 HUVEC cells per milliliter was then added to the 24-well plate and cultured in the incubator at 37 °C, 5% CO_2_, and 90% humidity. Images were captured at 2, 4, 6, and 8 h for observation. ImageJ software was utilized for image analysis and quantification.

### Chemotaxis and invasion assay

24-well plates and 8-μm-pore chemotaxis chambers (Falcon) were utilized in this experiment. Matrigel was diluted and plated on the polycarbonate membrane of the transwell chamber in the 24-well plate, with a volume of 50 μL per well. The plates were then polymerized into a gel at 37 °C for 30 min. Subsequently, 600 μL of 20% FBS medium was added to the 24-well plate, and 200 μL of cell suspension was added to the chambers at an appropriate concentration. After the incubation period, cells that did not pass through the chambers were washed away. The remaining cells were treated with PFA solution, 100% methanol solution, and a three-step staining kit. Three random fields were selected for imaging under a vertical microscope, and the number of cells passing through each field was counted for statistical analysis.

### Proteomic analysis

The experiment comprised two groups: the MHCC97H control group and the APOA2 overexpression group, each with three replicates. Proteomic analysis was conducted entirely by Shanghai Ji Kai Gene Chemical Technology Co., Ltd. For access to the original data and experimental procedures, readers can contact the corresponding author. Fisher’s Exact Test was employed in the enrichment analysis of GO or KEGG pathways on the target protein set to compare the distribution of each GO or KEGG pathway in the target protein set and the overall protein set, determining the significance level of protein enrichment in a GO term or KEGG pathway. Additionally, the correlation between the five highest expressed proteins (RPL22L1, ANAPC5, HEYL, RALYL, and CCDC33) and TGF-β in HCC was analyzed using the GEPIA website.

### TGF-β ELISA

TGF-β levels in mouse serum and cell culture medium were quantified using an ELISA method following the manufacturer’s instructions (Abcam, ab272386, USA).

### Real-time PCR

Total RNA was extracted from HCC cells and tissues using Trizol (Life Technologies) supplemented with glycogen (Ambion). Subsequently, reverse transcription was conducted, followed by real-time PCR analysis using the SYBR Green assay. The VEGFR2, Snail, and GAPDH primers were synthesized by BGI Bio-Solutions (Beijing, China) Co., Ltd., and the sequences of the primers used are listed in Supplementary Table 1.

### Glucose consumption and lactate production

The experiment was categorized into three groups: the MHCC97H control group, the APOA2 overexpression group, and the APOA2 overexpression + GW788388 (5 μM) group. Glucose consumption and lactate production of HCC cells in each group were quantified using the Automatic Biochemistry Analyzer (7170 A, Hitachi, Japan).

### Fuel oxidation analysis

The rate of cell oxidation of three mitochondrial fuels (glucose, glutamine, and long-chain fatty acids) was measured using the Mito Fuel Flex Test kit (Agilent, USA) following the manufacturer’s instructions.

### Analysis of fatty acid uptake

Cells were starved for 3 h, and then BODIPY™FLC16 (Invitrogen, D3821, USA) bound to bovine serum albumin was added. After incubation at 37 °C in a dark incubator for 30 min, the cells were washed with pre-cooled 1×PBS and separated by trypsinization. Finally, cells were fixed with 4% paraformaldehyde. Fatty acid uptake was determined by measuring intracellular fluorescence intensity using a Becton Dickinson flow cytometer Calibur and analyzed with cellular Quest Pro software (BD Biosciences, Germany).

### Measurement of FAO and cellular ATP

Cells were cultured in complete culture medium for 8 h followed by replacement with substrate-limiting medium for 4 h. Subsequently, the cells were replaced with FAO assay medium and incubated at 37 °C for another 45 min. Prior to the assay, 30 µl of palmitic-BSA substrate or control BSA and 10 µl of recombinant MitoXpress^®^ reagent were added to the wells. The OCR was measured using the Envision^®^ multimode tablet reader (Perkin Elmer, USA) and the MitoXpress^®^ Xtra oxygen consumption assay kit (Luxcel Bioscience, Ireland), with the slope of the fluorescence curve representing the OCR value. Intracellular ATP levels were measured using the CellTiter-Glo 2.0 Assay kit (Promega, USA) according to the manufacturer’s instructions.

### Statistics

GraphPad Prism 8.0 (provided by La Jolla, CA, USA) was used for statistical analyses, and the values were expressed in the form of mean ± SD. Two groups with the data obeying normal distribution were compared by *t*-test, and Analysis of Variance (ANOVA), along with Tukey’s test, was used for the comparison among several groups.

*P* < 0.05 was considered statistically significant. “^∗^means *P* < 0.05,” “^∗∗^means *P* < 0.01,” “^∗∗∗^means *P* < 0.001.”

## Supplementary information

Supplementary Table 1

Supplementary Table 2

Full uncropped Gels and Blots image(s)

## Data Availability

All data relevant to the study are included in the article or uploaded as online supplementary information.
