# Supplementary material for: APOA2-mediated endothelial mesenchymal transition and cancer lipid metabolism reprogramming confers antiangiogenic drug resistance through TGF-β
Source: Cell Death Discov. 2026 Feb 27;12:119. doi: 10.1038/s41420-026-02984-5 (PMC13004997; doi:10.1038/s41420-026-02984-5)
Supplement: Supplementary file 1 — Supplementary Table 1 [file 41420_2026_2984_MOESM1_ESM.docx]

**Supplementary Table 1. Primers used in real-time PCR.**

| **Gene** | **Forward** | **Reverse** |
| --- | --- | --- |
| VEGFR2 | GGAAATGACACTGGAGCCTA | TTTGAAATGGACCCGAGACA |
| Snail | AATCGGAAGCCTAACTACAGCGAG | CCTTG GCCTCAGAGAGCTGG |
| GAPDH | GTCTCCTCTGACTTCAACAGCG | ACCACCCTGTTGCTGTAGCCAA |
