# Supplementary material for: APOA2-mediated endothelial mesenchymal transition and cancer lipid metabolism reprogramming confers antiangiogenic drug resistance through TGF-β
Source: Cell Death Discov. 2026 Feb 27;12:119. doi: 10.1038/s41420-026-02984-5 (PMC13004997; doi:10.1038/s41420-026-02984-5)
Supplement: Supplementary file 2 — Supplementary Table 2 [file 41420_2026_2984_MOESM2_ESM.docx]

**Supplementary Table 2 Clinical information for all patients.**

| Resistance | | | | Sensitive | | | |
| --- | --- | --- | --- | --- | --- | --- | --- |
| AFP | | Volume | | AFP | | Volume | |
| Before | After | Before | After | Before | After | Before | After |
| 58868 | 194234 | 1433.5788 | 2607.395 | 17718 | 14.78 | 2.56 | 0.2 |
| 416 | 1233 | 945.14 | 2147.98 | 568 | 6 | 1048.22 | 456.12 |
| 562 | 4871 | 978.46 | 2298.36 | 987 | 48 | 946.56 | 411.78 |
| 2340 | 1894 | 489.89 | 2356.78 | 1246 | 98 | 979.36 | 945.17 |
| 460 | 20100 | 841.98 | 2897.41 | 25986 | 1245 | 5478 | 1842.10 |
| 789 | 2487 | 1478.22 | 3601.23 | 12498 | 456 | 2143.88 | 846.32 |
| 120 | 999 | 1568.29 | 3210.45 | 3698 | 301 | 1948.14 | 786.42 |
| 1491 | 6980 | 1425.79 | 4658.21 | 2879 | 254 | 1786.46 | 578.68 |
| 457 | 7320 | 2882.29 | 3749.18 | 34120 | 478 | 1988.46 | 555.48 |
| 98 | 631 | 2417.58 | 5918.97 | 126 | 19 | 3412.56 | 1478.64 |
