# Supplementary material for: APOA2-mediated endothelial mesenchymal transition and cancer lipid metabolism reprogramming confers antiangiogenic drug resistance through TGF-β
Source: Cell Death Discov. 2026 Feb 27;12:119. doi: 10.1038/s41420-026-02984-5 (PMC13004997; doi:10.1038/s41420-026-02984-5)
Supplement: Supplementary file 3 — Full uncropped Gels and Blots image(s) [file 41420_2026_2984_MOESM3_ESM.pdf]

Fig2c

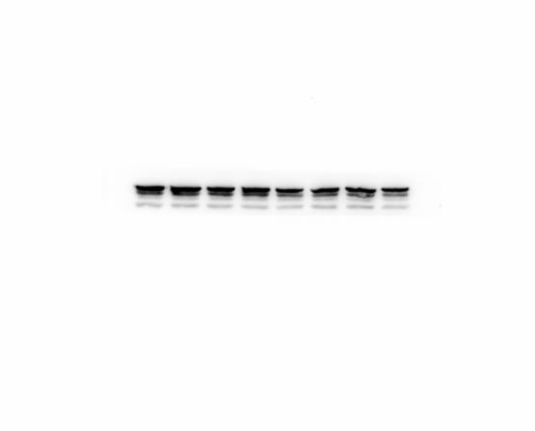

Actin

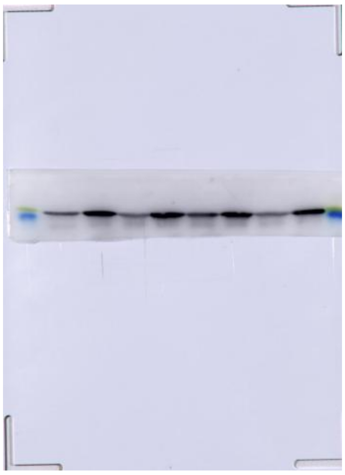

APOA2

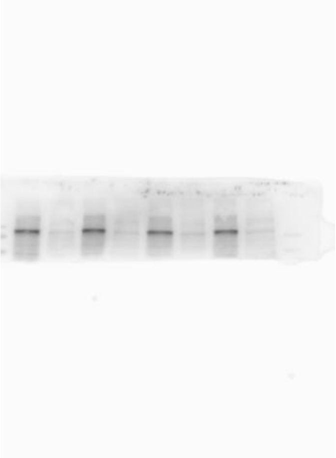

VEGFR2

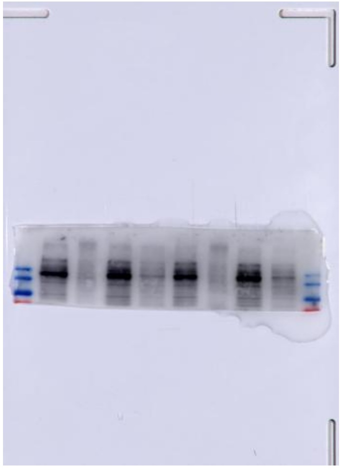

P-VEGFR2

Fig2d

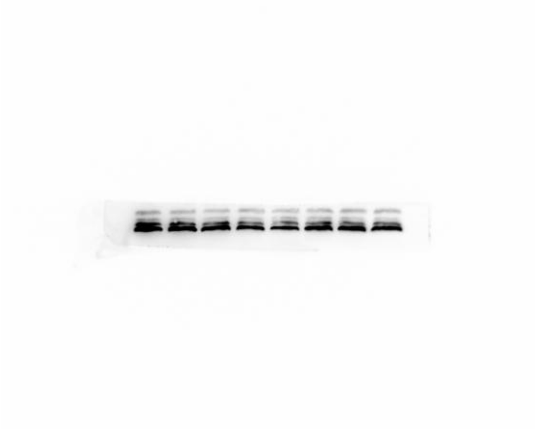

Actin

APOA2

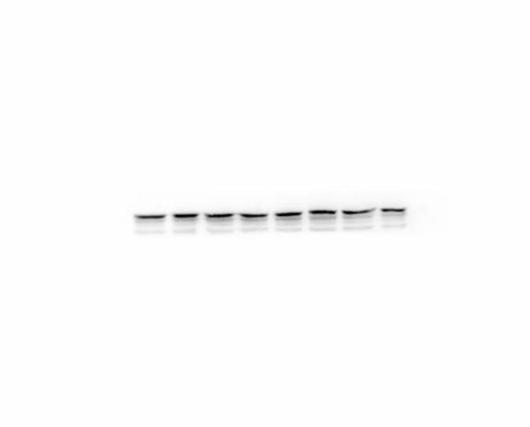

VEGFR2

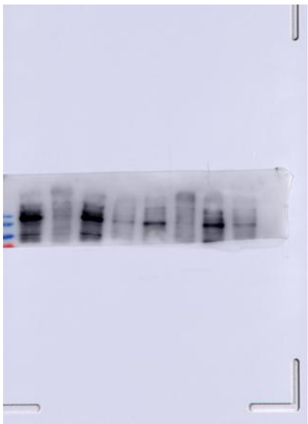

P-VEGFR2

Fig3

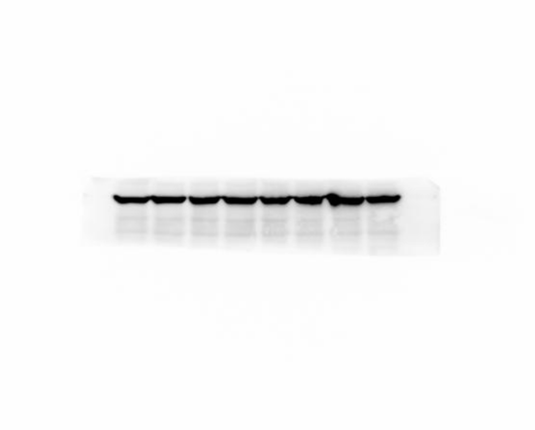

Actin

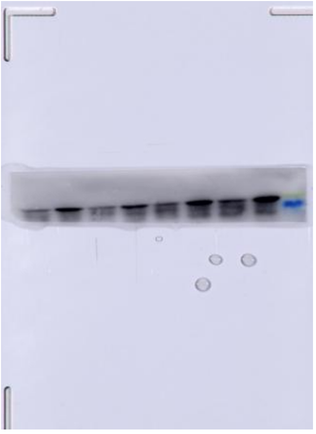

APOA2

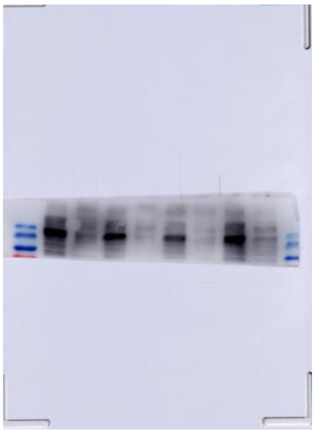

VEGFR2

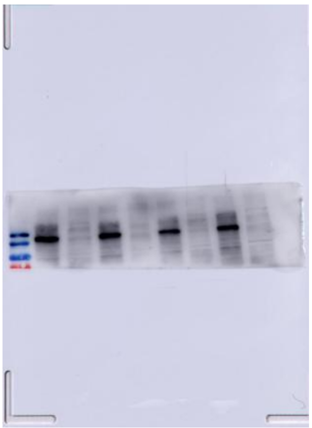

P-VEGFR2

Fig6h

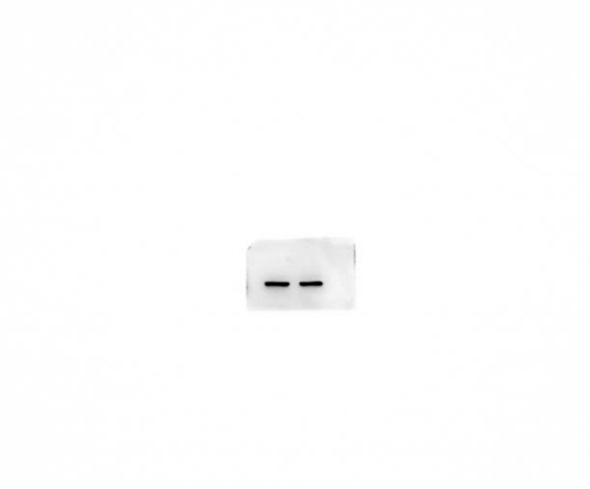

MHCC97H-β-Actin

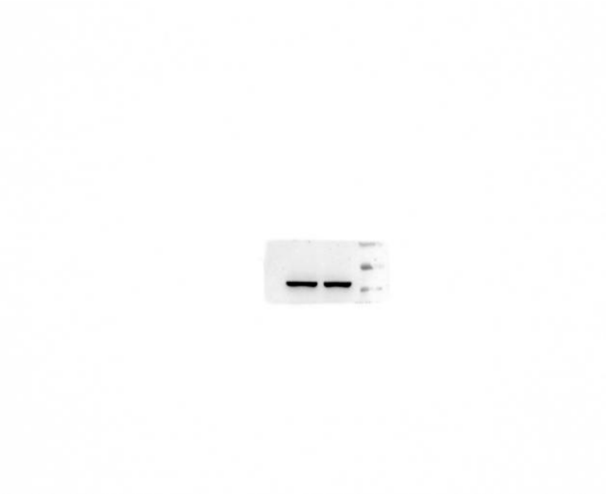

Tumor tissue-β-Actin

MHCC97H&Tumor tissue-TGFβR2

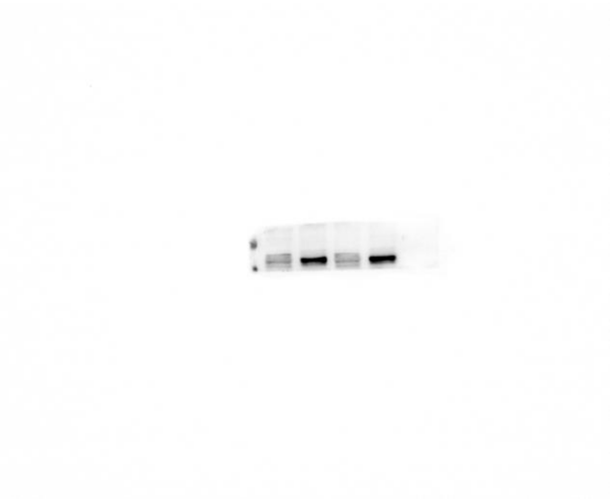

MHCC97H&Tumor tissue-P-TGFβR2

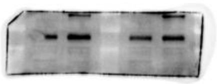

MHCC97H&Tumor tissue-P-TGFβR1

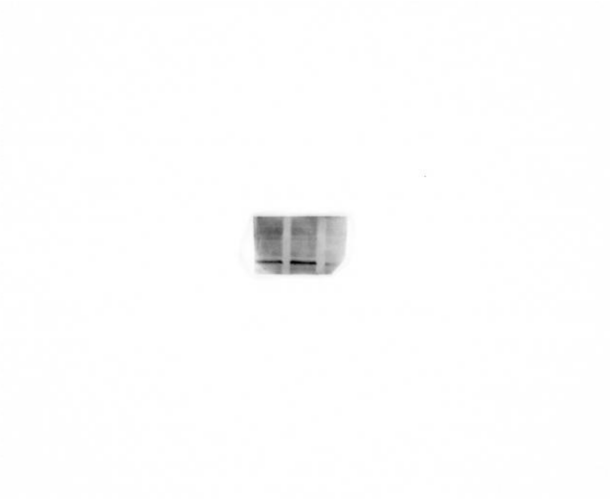

MHCC97H-P-TGFβR1

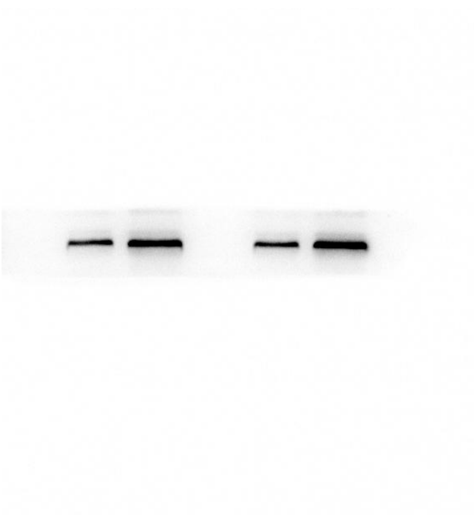

MHCC97H&Tumor tissue-TGFβR1

Fig8a

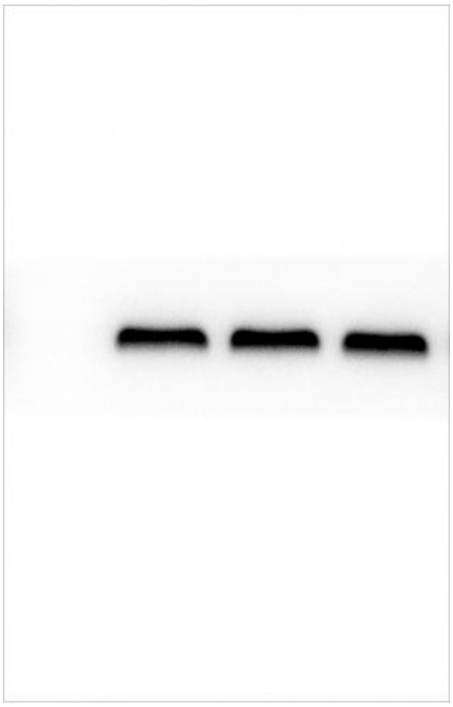

β-Actin

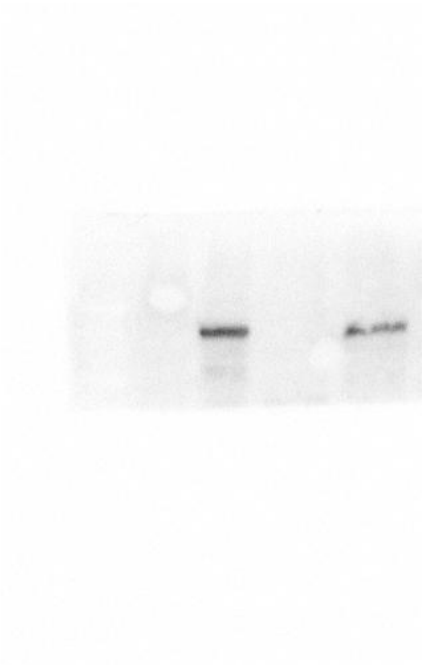

VEGFR2

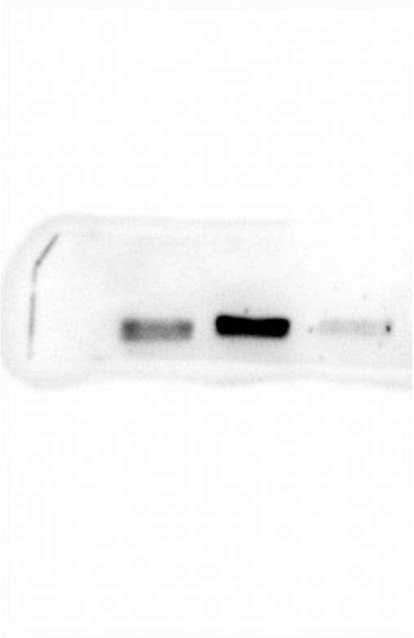

N-cadherin

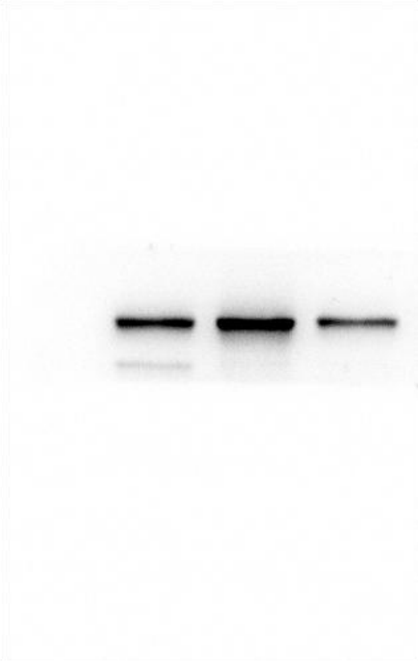

Snail
